# Machine learning combined multi-omics analysis to explore key oxidative stress features in systemic lupus erythematosus

**DOI:** 10.3389/fimmu.2025.1567466

**Published:** 2025-06-20

**Authors:** Hongwei Zhou, Xiaoqing Li, Yanyu Zhang, Feng Wei, Zhiyu Liu, Yan Zhao, Xubo Zhuang, Xia Liu, Haizhou Zhou

**Affiliations:** Department of Laboratory Diagnosis, The First Affiliated Hospital of Harbin Medical University, Harbin, Heilongjiang, China

**Keywords:** systemic lupus erythematosus, oxidative stress, metabolomics, transcriptomics, single-cell transcriptomic, machine learning

## Abstract

**Objective:**

Metabolic dysregulation and redox imbalance in immune cells are key drivers of systemic lupus erythematosus (SLE) pathogenesis. This study explores critical oxidative stress (OS) features and their interrelationships in SLE pathogenesis.

**Methods:**

Three transcriptomic datasets from the Gene Expression Omnibus (GEO) were analyzed to identify SLE- and OS-associated pathways via Gene Set Variation Analysis (GSVA). Multiple machine learning methods—including deep learning (DL), random forest (RF), XGBoost, support vector machine (SVM), and least absolute shrinkage and selection operator (LASSO)—were deployed to build OS-related gene prediction frameworks. Immune infiltration was assessed using CIBERSORT, and single-cell transcriptomic data from GEO elucidated gene expression patterns in various immune cell subsets. Peripheral blood plasma samples from confirmed SLE patients and healthy controls (HC) were analyzed using liquid chromatography-mass spectrometry (LC-MS) for metabolomics profiling and to evaluate OS and antioxidant stress (AOS) levels. Finally, real-time quantitative PCR (RT-qPCR) was used to validate the expression differences of key genes in peripheral blood mononuclear cells (PBMCs) from SLE patients and HC.

**Results:**

GSVA identified 15 metabolic pathways significantly linked to SLE, seven of which were strongly associated with OS and energy metabolism. LC-MS revealed substantial alterations in serum OS-related metabolites, clearly distinguishing SLE patients from healthy controls. A comprehensive machine learning approach pinpointed 10 OS-related genes; among these, six (ABCB1, AKR1C3, EIF2AK2, IFIH1, NPC1, SCO2) showed robust predictive performance and significant correlations with immune cell subsets. Single-cell analysis confirmed these genes’ expression in diverse immune cell types, consistent with the observed metabolic pathway disruptions. RT-qPCR verified downregulation of ABCB1, AKR1C3, and NPC1 and upregulation of EIF2AK2, IFIH1, and SCO2 in SLE PBMCs. SLE patients exhibited higher OS levels and lower AOS levels. Correlation analysis underscored strong relationships among key genes, OS/AOS levels, and vital metabolites.

**Conclusion:**

This multi-omics and machine learning–based investigation uncovered major disruptions in OS-related metabolic pathways and metabolites in SLE, ultimately identifying six key genes with distinct expression patterns across immune cell subsets. Their strong associations with OS/AOS levels and crucial metabolites highlight their diagnostic and therapeutic potential, laying a foundation for early detection and targeted treatment strategies.

## 1 Introduction

Systemic Lupus Erythematosus (SLE) is an autoimmune disease marked by heightened T and B cell activity and excessive production of autoantibodies that attack healthy tissues and cells (1). Emerging evidence highlights a close relationship between metabolites and the onset and progression of autoimmune diseases. For example, Luan et al. identified 26 blood metabolites as biomarkers for rheumatoid arthritis using metabolomic analysis. (2). Similarly, Li et al. applied Serum Metabolic Fingerprinting (SMF) to identify four metabolites capable of distinguishing SLE patients from healthy controls, validating their diagnostic potential through machine learning algorithms (3). In addition to metabolic dysregulation, redox imbalance is a hallmark of SLE (4). Reactive Oxygen Species (ROS), a key indicator of cellular redox imbalance, have been shown to correlate strongly with disease activity in SLE patients (5). Metabolic pathways play a pivotal role in modulating redox levels by providing substrates and regulating electron transfer (6, 7). For instance, mitochondrial oxidative phosphorylation generates ATP but simultaneously produces substantial amounts of ROS (8), while glutathione scavenges ROS, shielding cells from oxidative stress (OS) damage (9). Consequently, growing evidence suggests that OS and metabolism-related indicators may serve as promising diagnostic biomarkers and therapeutic targets for SLE.

Previous studies have highlighted the intricate associations between SLE, OS, and metabolic abnormalities. Aberrant activation of the glycolytic pathway in T cells of SLE patients has been shown to promote an increase in Th17 cells while suppressing Treg differentiation. This dysregulation activates multiple inflammatory pathways and amplifies the release of inflammatory cytokines (10–12). Additionally, CD4+ T cells in SLE patients exhibit elevated phosphofructokinase activity, which correlates significantly with disease activity, as measured by the SLEDAI score. Targeting phosphofructokinase (PFKP) using the CaMK4 inhibitor KN93 or CRISPR-Cas9-mediated knockdown reduces glycolytic activity and OS levels. These interventions enhance Treg functionality and stability, thereby mitigating SLE-like disease manifestations (13). Furthermore, monocytes from SLE patients undergo metabolic reprogramming characterized by simultaneous upregulation of glycolysis and oxidative phosphorylation. This metabolic shift is closely associated with elevated levels of type I interferon-alpha (IFNα) stimulation, a hallmark feature in SLE pathogenesis (14).

**Figure 1:**
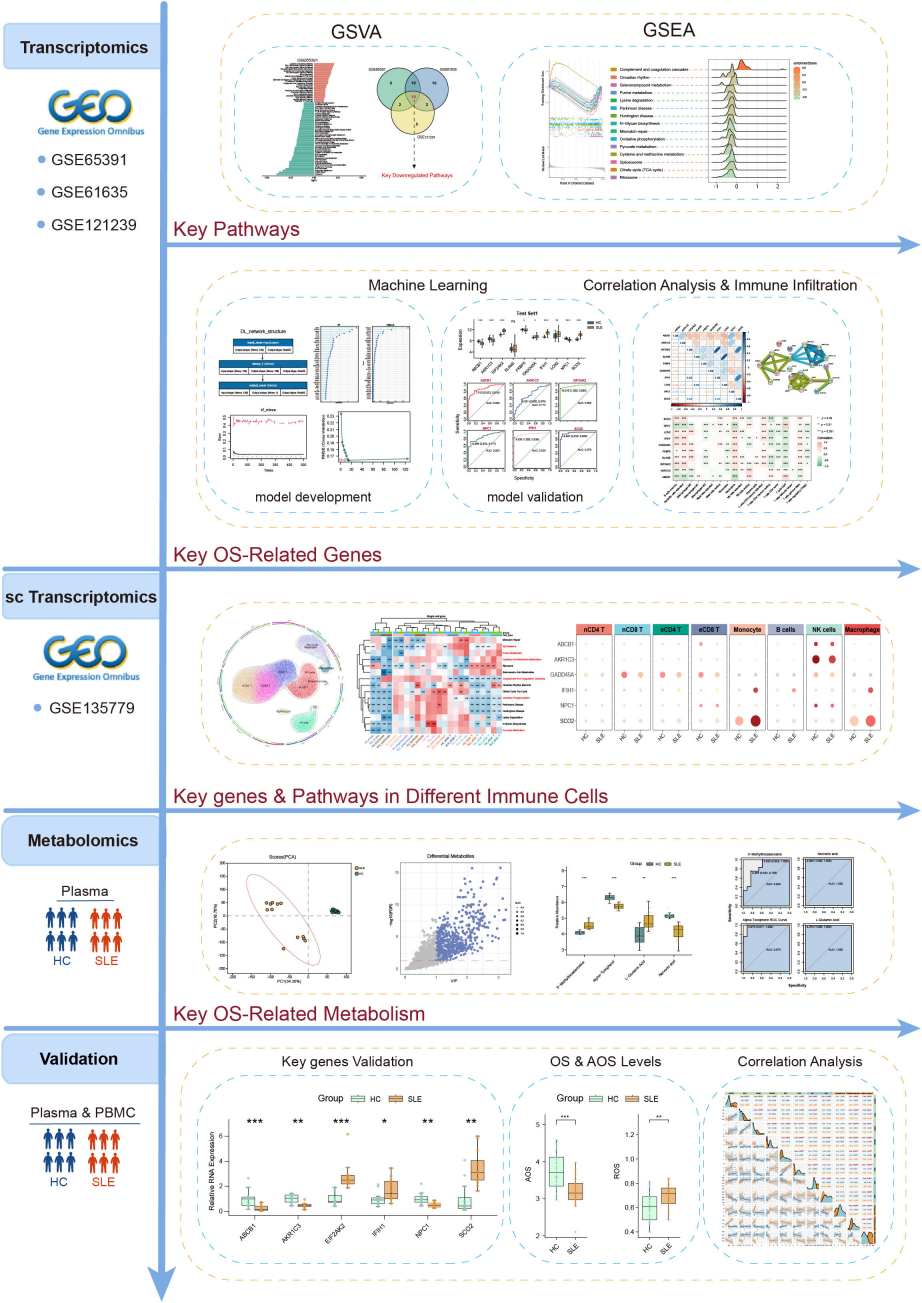
The flowchart illustrating the investigation procedure of this study.

The above studies underscore the importance of exploring key OS mechanisms and associated metabolic pathways in the progression of SLE. Such analyses not only deepen our understanding of SLE pathogenesis but also offer valuable insights for clinical diagnosis and treatment. However, comprehensive multi-omics integration focusing specifically on OS—combined with stringent validation and advanced algorithmic modeling—remains limited. To address these gaps, this study utilized a multi-omics approach combined with various machine learning algorithms to systematically unravel the intricate network relationships among OS-related metabolic pathways, metabolites, and key genes in SLE. The detailed workflow is illustrated in **Figure 1**. Gene Set Variation Analysis (GSVA) was conducted on multiple SLE transcriptomic datasets, alongside Liquid Chromatography-Mass Spectrometry (LC-MS)-based metabolomics, to identify OS-related pathways and metabolites. Five distinct machine learning methods were then employed to construct and evaluate predictive models for SLE, leading to the identification of 10 key feature genes. Immune infiltration analysis and single-cell transcriptomic data further validated the expression patterns and functional states of these genes across various immune cell subsets. Finally, OS and antioxidant stress (AOS) levels were measured. Subsequently, real-time quantitative polymerase chain reaction (RT-qPCR) was used to validate the expression of key genes, aiming to explore their intrinsic relationships in SLE patients versus healthy controls. This comprehensive analysis revealed intrinsic relationships among OS, AOS, and metabolic pathways, providing novel theoretical insights into SLE pathogenesis and establishing a foundation for future diagnostic and therapeutic innovations.

## 2 Methods

### 2.1 Data sources

Bulk transcriptome microarray datasets (GSE65391, GSE61635, and GSE121239) and single-cell RNA-seq dataset (GSE135779) from peripheral blood samples of SLE patients and healthy controls were obtained from the Gene Expression Omnibus (GEO, https://www.ncbi.nlm.nih.gov/geo/). Details of these datasets are provided in **Table 1**. A comprehensive list of OS-related genes was curated from GeneCards (https://www.genecards.org/) by searching with the keyword “oxidative stress” and applying a relevance score threshold greater than 7, following previously described methods, yielding a total of 1745 genes (15–17).

**Table 1:** Summary of the datasets used in this study.

Dataset	GPL platform	Sample		Sample type	Age median (Q1, Q3)	SLEDAI (SLE Patients) median (Q1, Q3)	Year	Data link
		SLE	HC					
GSE65391	GPL10558	924	72	Whole blood	14.8 (12.7, 16.8)	4 (2, 8)	2016	https://www.ncbi.nlm.nih.gov/geo/query/acc.cgi?acc=GSE65391
GSE61635	GPL570	99	30	Whole blood	Not reported	Not reported	2015	https://www.ncbi.nlm.nih.gov/geo/query/acc.cgi?acc=GSE61635
GSE121239	GPL13158	292	20	PBMC	47 (37, 55)	2 (0, 4)	2018	https://www.ncbi.nlm.nih.gov/geo/query/acc.cgi?acc=GSE121239
GSE135779	GPL20301	40	16	PBMC	17 (14, 18)	4 (2, 6)	2020	https://www.ncbi.nlm.nih.gov/geo/query/acc.cgi?acc=GSE135779

### 2.2 Bulk transcriptomic analysis

#### 2.2.1 Pathway-level feature identification

GSVA was performed using the gsva function in the GSVA package (version 1.53.4) in R on the gene expression matrix. Pathway information from the KEGG gene sets in the Molecular Signatures Database (MSigDB, version 7.5.1; category C2, subcategory KEGG) (18) was used to construct the pathway sets. The limma package (version 3.60.6) was then applied to extract the GSVA enrichment scores for each sample and conduct differential analysis. Metabolic pathways with an adjusted p-value (adj.P.Val) < 0.05 and |Log2FC| > 0.1 were considered significantly different. Gene Set Enrichment Analysis (GSEA) was also conducted using the clusterProfiler package (version 4.12.6) to further evaluate pathway-level enrichment patterns.

#### 2.2.2 Feature selection, model construction and validation

Using the limma package, differentially expressed genes (adj.P.Val < 0.05, |log2FC| ≥ 0.5) between SLE patients and healthy controls in the GSE65391 dataset were identified, yielding a total of 1,536 differentially expressed genes. The resulting gene set was then intersected with OS-related genes obtained from GeneCards, yielding OS-related differential genes. These OS-related differential genes were subsequently used for machine learning model training. Specifically, dataset GSE65391 served as the training set, while GSE61635 and GSE121239 served as test sets 1 and 2, respectively, for model development and validation.

A three-layer neural network was implemented as a deep learning (DL) model using the R package tensorflow (version 2.16.0). The network included an input layer (124 genes), a hidden layer (128 neurons) with ReLU activation, and an output layer with a sigmoid activation function for binary classification. Network weights were initialized using a normal distribution (mean = 0, standard deviation = 0.05). The learning rate was 0.01, batch size was 128, and the model was trained for up to 600 iterations. Model performance was monitored by accuracy and loss during training, then evaluated via confusion matrix, sensitivity, and specificity after training. Gene importance was determined by extracting the model’s weight matrix and summing the absolute values of each gene’s weights. A random forest (RF) model was constructed using the randomForest package (version 2.16.0) with 500 decision trees (n_trees = 500). Gene importance was determined by the Gini index, selecting potential key genes. An XGBoost model was trained using the xgboost package (version 1.7.8.1) with a learning rate (eta) of 0.2, a maximum depth (max_depth) of 6, and 200 boosting rounds (nrounds = 200). Gene importance was ranked based on each tree’s contribution to reducing the loss function. Using the R packages caret (version 6.0-94), kernlab (version 0.9-33), and e1071 (version 1.7-16), Support Vector Machine-Recursive Feature Elimination (SVM-RFE) was conducted. Feature subset size ranged from 1–15 with a step of 2, and 10-fold cross-validation was employed to evaluate model performance. The optimal feature set was selected based on the subset that yielded the lowest root mean squared error (RMSE), and the most informative genes contributing to classification were identified. Least Absolute Shrinkage and Selection Operator (LASSO) regression was performed with the glmnet package (version 4.1-8), using 10-fold cross-validation to determine the penalty coefficient (λ). The optimal feature genes were selected based on the coefficient trajectory plot.

Key genes identified from the models were further validated using external datasets. Key gene expression differences were compared between the training set and test sets 1 and 2 via the Wilcoxon test. ROC analysis was performed using the pROC package (version 1.18.5). Protein–protein interactions among key genes were assessed via the STRING database (https://string-db.org/), and the Corrplot package (version 0.95) was used for correlation analysis.

#### 2.2.3 Immune infiltration

Immune infiltration was evaluated using the CIBERSORT algorithm, estimating the relative abundance of each immune cell subset in the transcriptomic data (19).

### 2.3 LC-MS serum metabolomics analysis

#### 2.3.1 Research subjects

Newly diagnosed SLE patients who met the 1997 American College of Rheumatology (ACR) diagnostic criteria for SLE were recruited from the Department of Rheumatology at the First Affiliated Hospital of Harbin Medical University between July 2023 and January 2025 (20). All patients/participants provided written informed consent to participate in this study. The exclusion criteria were as follows: (1) presence of acquired immunodeficiency syndrome (AIDS), active infections (including tuberculosis, hepatitis B, or hepatitis C), hypogammaglobulinemia, or a history of organ or hematopoietic stem cell transplantation; (2) diagnosis of overlapping autoimmune syndromes, including but not limited to mixed connective tissue disease (MCTD), Sjögren’s syndrome, systemic sclerosis, and rheumatoid arthritis; (3) history or presence of malignancies, including hematologic malignancies (e.g., lymphoma, leukemia) or solid tumors (e.g., lung, breast, or gastrointestinal cancers); and (4) incomplete clinical data, defined as missing essential information for disease activity assessment, such as complement levels (C3, C4), autoantibody profiles (e.g., ANA, anti-dsDNA), documentation of clinical manifestations, or recent medication history. Baseline demographic and clinical characteristics of the enrolled SLE patients and healthy controls are summarized in **Table 2**.

**Table 2:** Baseline characteristics of SLE patients.

Characteristic	HC (n=30)	SLE (n=30)	P,value
Age, median (Q1, Q3)	43.00 (31.25, 50.75)	40.50 (30.00, 50.00)	0.473
Gender (Male/Female)	2/28	2/28	1.000
BMI, median (Q1, Q3)	23.41 (20.64, 26.63)	23.21 (20.81, 25.45)	0.595
SLEDAI, median (Q1, Q3)	Not applicable	6.00 (2.00, 8.00)	Not applicable

#### 2.3.2 Serum sample collection and metabolite extraction

From a total of 60 clinical samples, 12 pairs of SLE patients and healthy controls were selected based on optimal matching of age, sex, and BMI to ensure comparability. These matched samples were subsequently used for LC-MS–based metabolomic analysis. Peripheral blood samples were collected from all participants and centrifuged at 3,700 rpm for 7 minutes at 4°C using a refrigerated centrifuge to obtain serum. All samples were processed on ice, and serum was separated within 30 minutes of collection to minimize oxidation. The resulting serum was immediately stored at −80°C until analysis. Before LC-MS analysis, 100 μL of serum was precisely transferred into a 1.5 mL centrifuge tube and mixed with 400 μL of extraction solution (acetonitrile: methanol = 1:1) containing 0.02 mg/mL L-2-chlorophenylalanine. After 30 s of vortexing, samples were sonicated at 5°C and 40 kHz for 30 minutes. The extract was then kept at −20°C for 30 minutes and centrifuged at 13,000 g and 4°C for 15 minutes; the supernatant was dried under nitrogen gas. The residue was redissolved in 100 μL of solvent (acetonitrile:water = 1:1) and sonicated under the same conditions for 5 minutes. After a subsequent 10-minute centrifugation at 13,000 g and 4°C, the supernatant was transferred into vials for LC-MS analysis.

#### 2.3.3 LC-MS analysis

Samples were analyzed on a Thermo Fisher UHPLC-Q Exactive HF-X system equipped with an HSST3 column. The injection volume was 3 μL. Mobile phase A was 95% water + 5% acetonitrile (containing 0.1% formic acid), and mobile phase B was 47.5% acetonitrile + 47.5% isopropanol + 5% water (containing 0.1% formic acid). The ion source was set to ESI+/ESI−, scanning over m/z 70–1050. Sheath gas was 50 psi, auxiliary gas was 13 psi, auxiliary gas heater temperature was 425°C, and spray voltages were +3500 V/−3500 V. The ion transfer tube was maintained at 325°C. Normalized collision energy (NCE) was set to 20–40–60 V. MS^1 and MS^2 resolutions were 60,000 and 7,500, respectively, and data-dependent acquisition (DDA) was employed to obtain metabolite information.

#### 2.3.4 Identification of metabolite species and data preprocessing

Raw data were preprocessed in Progenesis QI for peak extraction, alignment, and retention time correction, generating a data matrix characterized by retention time, m/z, and peak intensity. MS and MS/MS information were matched against HMDB, Metlin, and an in-house database for preliminary metabolite identification.

Variables with missing values greater than 20% within any experimental group were excluded, and remaining missing values were imputed with the minimum value across all samples. Subsequently, data normalization was conducted using sum normalization (total peak area) to correct for variability related to sample loading or injection volume differences. Features exhibiting a relative standard deviation (RSD) greater than 30% in QC samples were removed to ensure analytical reproducibility. Data were then transformed by log_10_ transformation to approximate a normal distribution.

Quality control (QC) samples were prepared by pooling equal volumes of extracts from all samples and injected periodically after every 5–15 samples during the LC-MS run. QC samples were evaluated by monitoring the total ion chromatogram (TIC) consistency and RSD values of peak intensities.

#### 2.3.5 Identification of differential metabolites

The R package ropls (version 1.6.2) was used for Principal Component Analysis (PCA) and Orthogonal Partial Least Squares-Discriminant Analysis (OPLS-DA). Model stability was evaluated via seven-fold iterative cross-validation. Metabolites meeting OPLS-DA VIP > 1, Student’s t-test p < 0.05, and ROC curve AUC > 0.7 were defined as significant differential metabolites.

### 2.4 Single-cell analysis

Single-cell data from GSE135779 were merged, yielding 59 samples (40 SLE, 16 HC) and a total of 295,200 cells. After filtering cells that had 200–2,000 genes expressed, the remaining data were normalized using the Seurat package. The ElbowPlot function was used to determine the dimensions for reduction, and highly variable genes were identified via FindVariableFeatures. UMAP was then applied for dimensionality reduction and cell type annotation. Further visualization was conducted using the plot1cell package (21).

### 2.5 Measurement of OS and AOS levels

A hydrogen peroxide kit (Comin Biotechnology, Suzhou, China) was used to measure H_2_O_2_ concentrations as an indicator of ROS levels. A total antioxidant capacity kit (ABTS method, Comin Biotechnology, Suzhou, China) was employed to determine AOS levels.

### 2.6 RT-qPCR experiments

#### 2.6.1 Isolation of peripheral blood mononuclear cells

Whole blood samples were mixed 1:1 with PBS, then carefully layered over an equal volume of Ficoll-Paque™ in a 15 mL centrifuge tube. Samples were centrifuged at 450 g and room temperature for 25 minutes. The white buffy coat (PBMCs)was gently transferred to a new 15 mL tube, resuspended in PBS, and centrifuged at 250 g for 10 minutes. After discarding the supernatant, 500 μL Trizol was added, and the suspension was stored in 1.5 mL EP tubes at −80°C.

#### 2.6.2 RT-qPCR

Each sample tube received 100 μL chloroform, was mixed thoroughly, and was left to stand for 5 minutes. The sample was then centrifuged at 12,000 g and 4°C for 15 minutes, and the top aqueous phase was transferred to a new tube. After adding 0.3 mL isopropanol and mixing, samples were kept at 4°C for 10 minutes and subsequently centrifuged at 12,000 g for 10 minutes. The supernatant was discarded, and the pellet was washed with 0.5 mL of 75% ethanol (prepared with DEPC), then centrifuged at 7,500 g and 4°C for 5 minutes. Following supernatant removal, the pellet was air-dried at room temperature for 3–5 minutes and dissolved in 20 μL RNase-free water to obtain the RNA solution.

An eight-tube strip was placed on ice, and each well received 2 μL of 5× RT Master Mix, 8 μL RNA solution, and 10 μL DEPC-treated water. After mixing, reverse transcription was performed at 37°C for 15 minutes followed by 85°C for 5 seconds, then held at 4°C to yield 20 μL cDNA. A 20 μL PCR reaction system was prepared according to the manufacturer’s protocol (F488 SYBR qPCR MIX 10 μL, cDNA 2 μL, forward primer 0.4 μL, reverse primer 0.4 μL, DEPC water 7.2 μL). Two-step PCR amplification was performed, and relative gene expression levels were calculated using the 2^−ΔΔCT^ method. GAPDH was used as the reference gene based on its previously reported stability in PBMCs (22). The sequences of all primers are listed in **Supplementary Table 1**.

### 2.7 Statistical analysis

All statistical analyses were performed using R software (version 4.4.1), with P < 0.05 considered statistically significant (*P < 0.05; **P < 0.01; ***P < 0.001). Data conforming to a normal distribution are presented as mean ± standard deviation (mean ± SD) and were compared using Student’s t-test or one-way ANOVA. Non-normally distributed data are expressed as median (interquartile range) [M (P25–P75)] and were analyzed using the Mann–Whitney U test. Correlation analyses were performed using Pearson’s correlation coefficient for normally distributed data or Spearman’s correlation coefficient for non-normally distributed data.

## 3 Results

### 3.1 Large-scale transcriptome analyses reveal metabolic pathway abnormalities in SLE patients

To systematically investigate potential metabolic pathway dysregulations in SLE patients, we first selected three transcriptomic datasets (GSE65391, GSE61635, and GSE121239) from the GEO database. Based on 186 metabolic pathways cataloged in the KEGG database (involving 16,283 human genes related to metabolism), GSVA was performed. By analyzing differences in metabolic pathway activity across each dataset, we aimed to identify key metabolic pathways closely tied to SLE pathogenesis.

**Figure 2:**
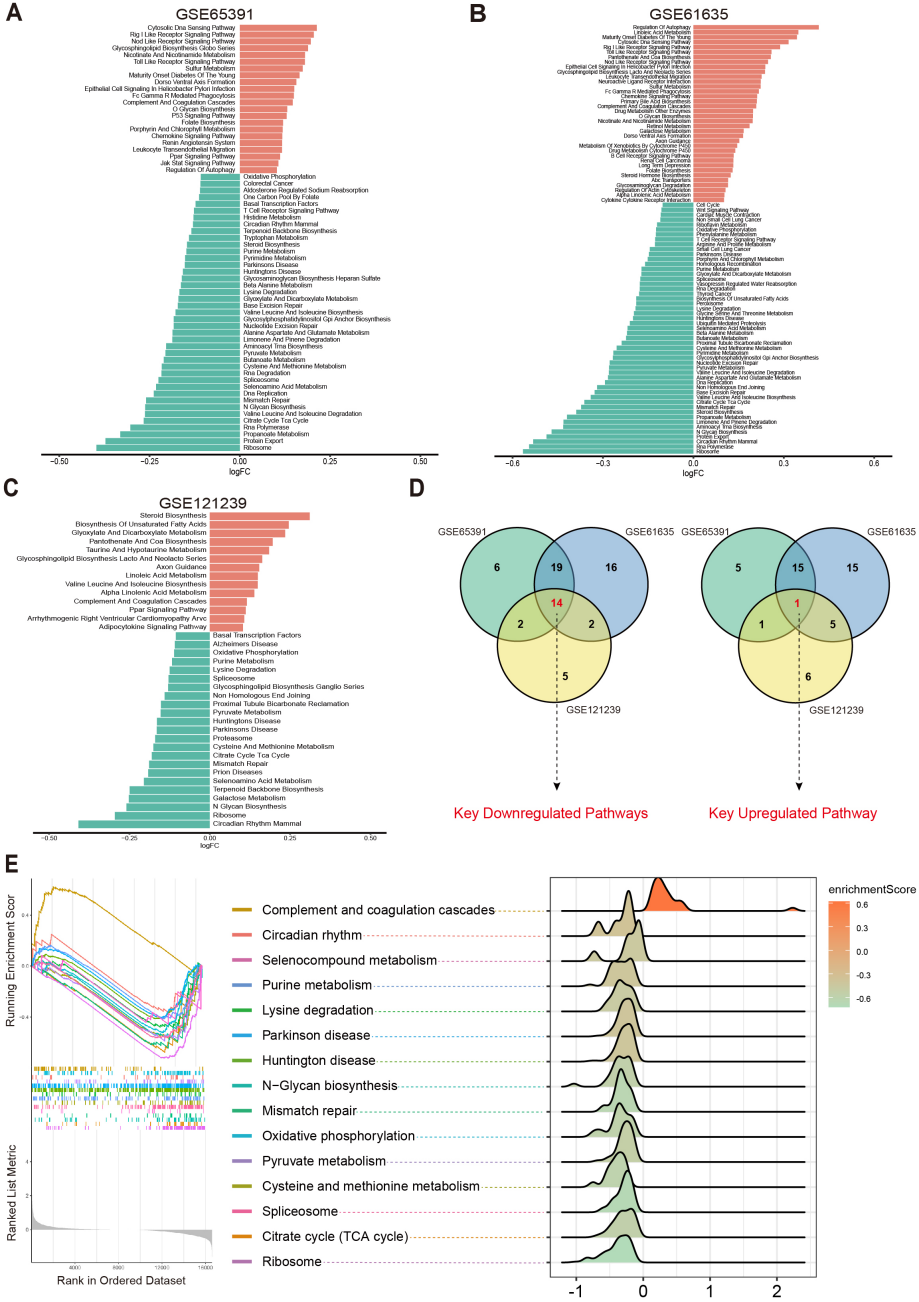
Differential metabolic pathways in SLE patients. **(A–C)** Bar plots displaying the differential metabolic pathways in SLE patients across different datasets, with orange bars representing upregulated pathways and green bars representing downregulated pathways. **(D)** Venn diagrams showing the key downregulated and key upregulated metabolic pathways. **(E)** GSEA enrichment plots and ridge plots providing additional insights into the alterations of these key pathways in SLE.

The results showed that in the GSE65391 dataset, 41 pathways were downregulated and 22 pathways were upregulated (**Figure 2A**); in GSE61635, 51 pathways were downregulated and 36 pathways were upregulated (**Figure 2B**); and in GSE121239, 23 pathways were downregulated and 14 pathways were upregulated (**Figure 2C**). Subsequently, a Venn diagram was used to merge the upregulated and downregulated pathways from each dataset, ultimately yielding 15 key metabolic pathways that were differentially expressed across multiple datasets: Circadian Rhythm Mammal, Ribosome, N Glycan Biosynthesis, Selenoamino Acid Metabolism, Mismatch Repair, Citrate Cycle (TCA Cycle), Cysteine and Methionine Metabolism, Parkinson’s Disease, Huntington’s Disease, Pyruvate Metabolism, Spliceosome, Lysine Degradation, Purine Metabolism, Oxidative Phosphorylation, and Complement and Coagulation Cascades (**Figure 2D**).

Further analysis revealed that Oxidative Phosphorylation, Cysteine and Methionine Metabolism, Purine Metabolism, Pyruvate Metabolism, Citrate Cycle (TCA Cycle), Spliceosome, and Complement and Coagulation Cascades were all closely associated with OS and cellular energy metabolism (23–27). Subsequent GSEA confirmed the abnormal changes in these key pathways among SLE patients (**Figure 2E**). Taken together, compared with healthy controls (HC), SLE patients exhibited significant differences across multiple metabolic pathways, particularly those related to OS and cellular metabolic processes.

### 3.2 LC-MS metabolomics indicates altered OS-related key metabolites in SLE patients

**Figure 3:**
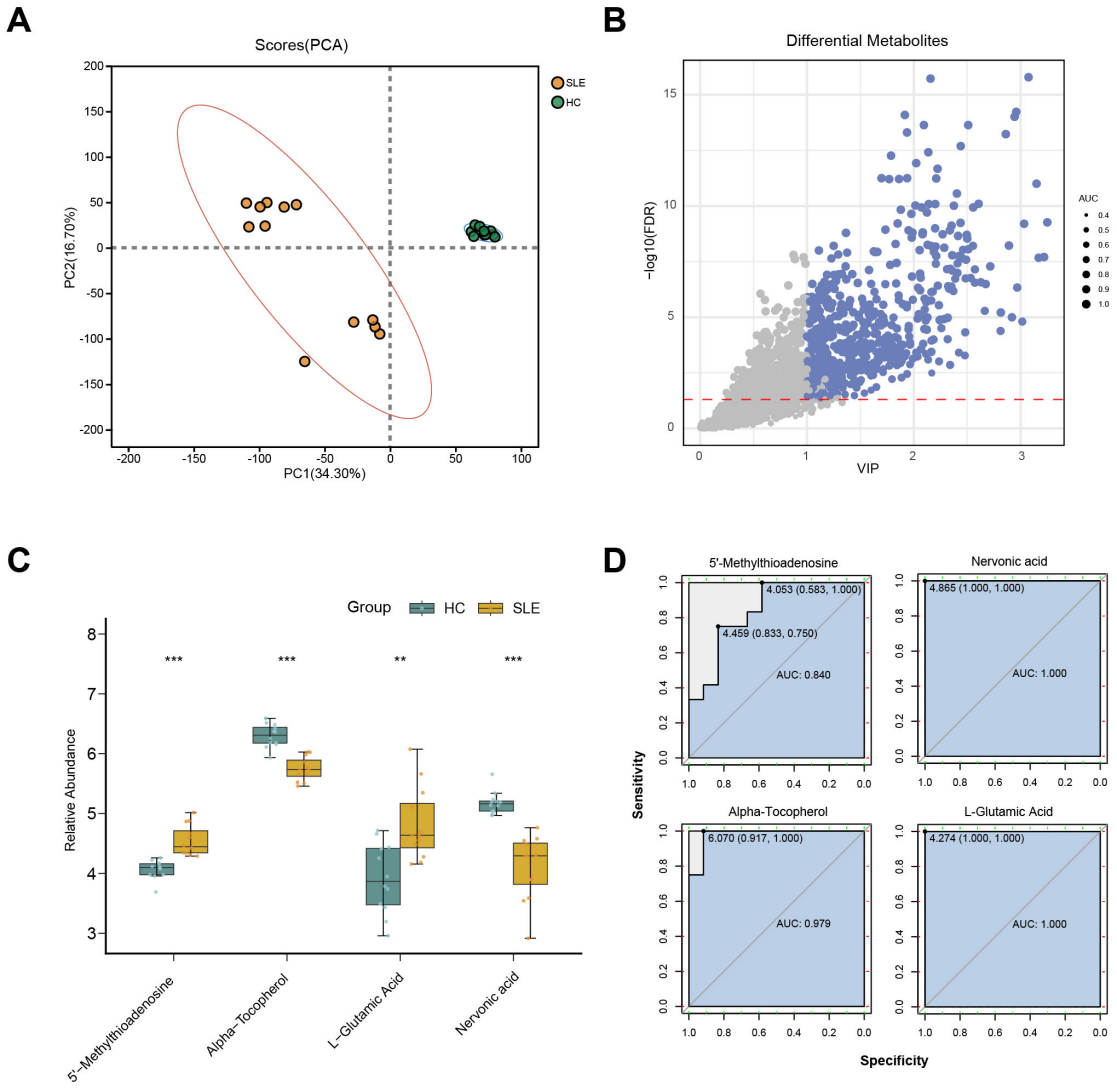
LC-MS metabolomics in SLE patients versus healthy controls. **(A)** PCA scatterplot illustrating distinct overall metabolic patterns in the SLE group (brown circles) compared with the healthy control group (green circles). **(B)** Volcano plot of differential metabolites, where the x-axis denotes VIP values and the y-axis denotes −log10(p-value). Circle size corresponds to AUC values; blue circles indicate selected differential metabolites. **(C)** Boxplots of key OS-related metabolites in SLE patients versus healthy controls. (**P < 0.01, ***P < 0.001). **(D)** ROC curves assessing the diagnostic performance of these key metabolites in distinguishing SLE from healthy controls.

To further validate potential abnormalities in OS-related metabolic pathways in SLE, we conducted LC-MS–based metabolomics on serum samples from SLE patients and HCs. A total of 2,127 metabolites were detected, and PCA revealed that the overall metabolic profiles of SLE and HC groups were markedly distinct; moreover, intra-group variation among SLE patients was higher than in the HC group (**Figure 3A**). This finding suggests that SLE patients exhibit not only conspicuous differences from healthy individuals in terms of metabolite composition but also substantial heterogeneity within their own metabolic characteristics.

Further differential metabolite screening combined with ROC analysis identified 577 metabolites as significantly altered (p < 0.05, VIP > 1, AUC > 0.7) (**Figure 3B**, **Supplementary Table 2**). Among these, key metabolites closely related to OS—such as L-glutamate, 5′-methylthioadenosine, α-tocopherol, and L-nervonic acid (28–30)—showed significant changes in SLE patients (**Figure 3C**). In addition, ROC analysis indicated strong predictive power for these metabolites (**Figure 3D**). Taken together, the LC-MS metabolomics results provide further support for the presence of OS-associated metabolic disturbances in SLE.

### 3.3 Identification of OS-related key genes in SLE via machine learning

To identify SLE-related genes associated with OS, we intersected the differentially expressed genes from the training set with the OS-related gene set curated from GeneCards. This yielded 124 OS-related differentially expressed genes, which were subsequently used for model training and feature selection.

**Figure 4:**
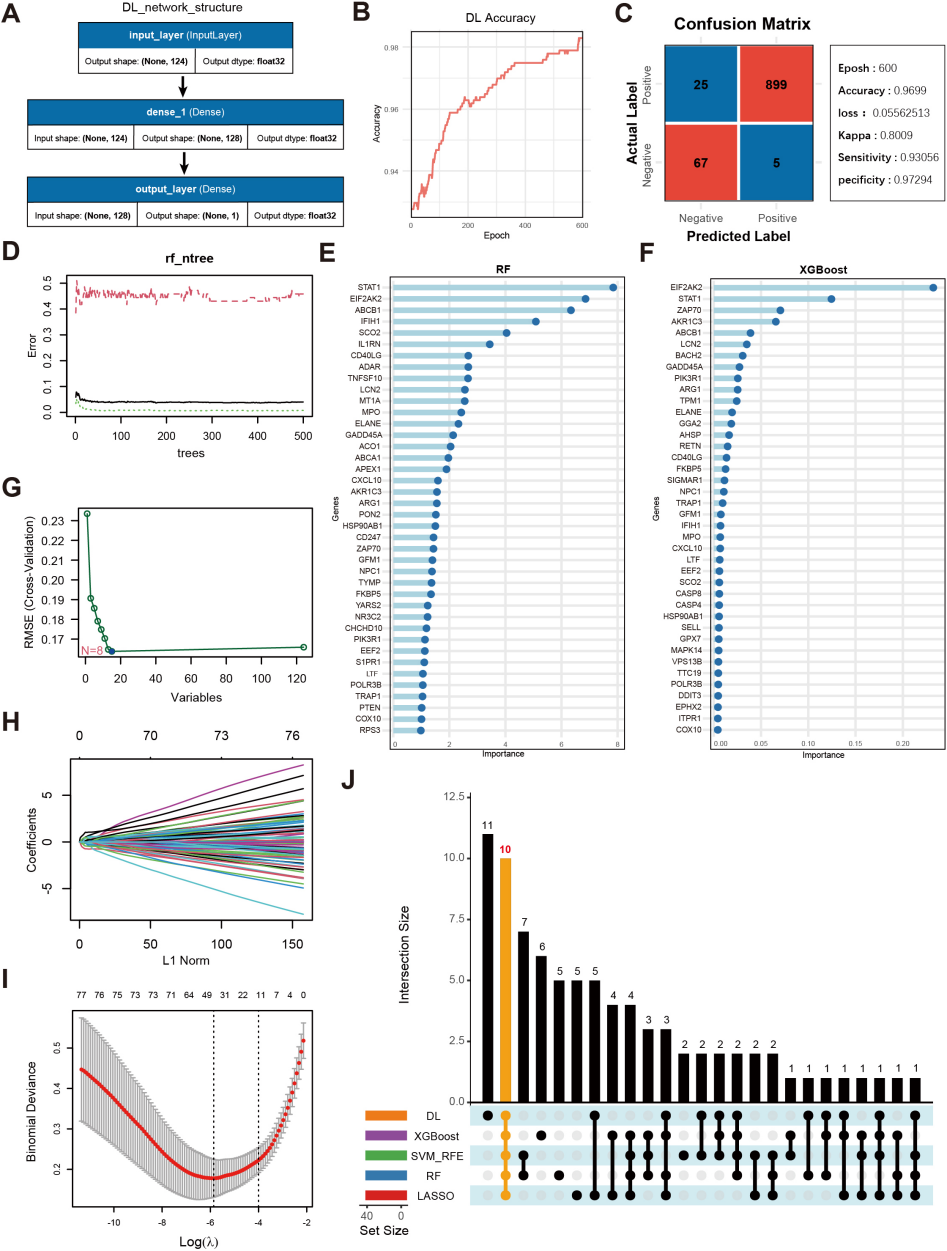
Screening of key genes using multiple machine learning algorithms and model performance evaluation. **(A)** Schematic diagram of the DL model architecture, consisting of the input layer and two fully connected layers (Dense, output_layer). The input layer receives 124 genes as features, culminating in a final prediction at the output layer. **(B)** Accuracy trend of the deep learning model over the training process. **(C)** Confusion matrix visualization of the deep learning model and main performance metrics. “Epoch” denotes the number of training iterations. “Accuracy” is the final accuracy, “Kappa” is the coefficient for consistency testing, and “Sensitivity” and “Specificity” represent the model’s sensitivity and specificity, respectively. **(D)** Trend plot of RF model error versus the number of decision trees. **(E)** Gene importance rankings identified by the RF model, where the x-axis shows importance scores and the y-axis lists genes. **(F)** Gene importance ranking from the XGBoost model, with the x-axis showing importance scores and the y-axis listing genes. **(G)** Selection of key OS-related genes using SVM-RFE. The x-axis indicates the number of feature genes, and the y-axis represents the cross-validation outcome. **(H)** Trajectory of coefficients in LASSO regression as the regularization strength (L1Norm) changes. **(I)** Cross-validation error curve in the LASSO model, with vertical lines indicating possible optimal λ ranges. **(J)** UpSet plot displaying the overlapping sets of important genes identified across the five machine learning models.

**Figure 4A** displays a schematic of the neural network model; after 600 iterations, the model’s accuracy stabilized above 0.98 (**Figure 4B**), and a confusion matrix was used to visualize the model’s performance (**Figure 4C**). To further validate the model’s robustness and the accuracy of the identified key genes, we also employed RF (**Figures 4D,E**), XGBoost (**Figure 4F**), SVM (**Figure 4G**), and LASSO regression (**Figures 4H–I**) to analyze feature importance for the same gene set. The top 40 OS-related genes identified by each method were selected based on model performance and feature ranking. By integrating the results from the five machine learning models, 10 genes consistently demonstrated high feature importance across all methods (**Figure 4J**): ABCB1, AKR1C3, EIF2AK2, ELANE, FKBP5, GADD45A, IFIH1, LCN2, NPC1, and SCO2.

### 3.4 Relationships among key genes and their predictive performance in training and test sets

**Figure 5:**
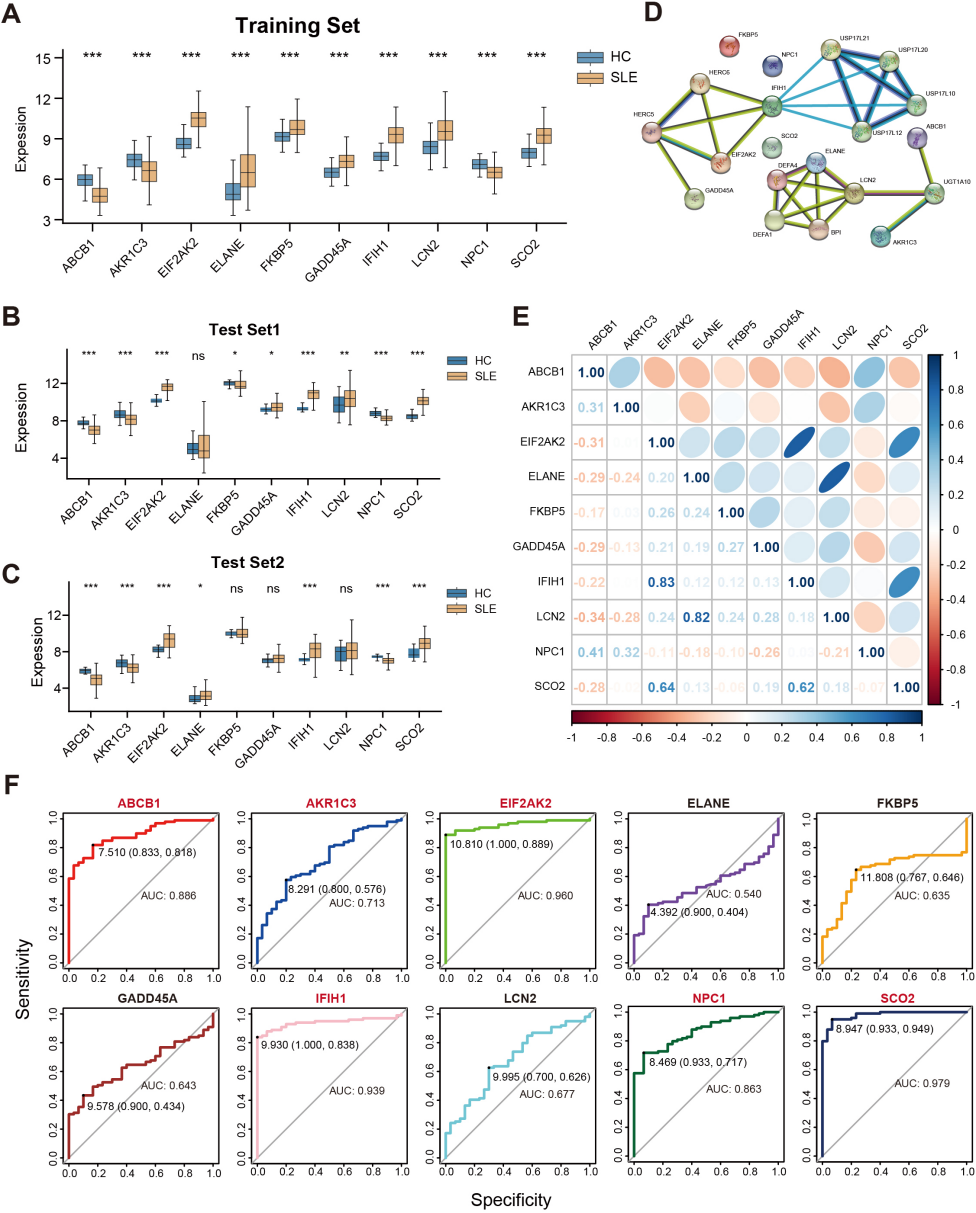
Correlations among key genes and their expression differences in the training and test sets. **(A)** Boxplots of the relative expression levels for 10 key genes in SLE patients versus HCs in the training set. **(B, C)** Boxplots of the 10 key genes in Test Sets 1 and 2. **(D)** Protein–protein interaction network illustrating the interactions among proteins corresponding to these key genes. **(E)** Pearson correlation matrix of the key genes. Color intensity indicates the absolute value of the correlation coefficient (blue for positive correlation, orange for negative correlation). **(F)** ROC curves evaluating the ability of the 10 key genes to distinguish SLE from HCs in the validation set, where the x-axis indicates specificity and the y-axis indicates sensitivity. (ns” indicates “not significant, *P < 0.05, **P < 0.01, ***P < 0.001).

We conducted a systematic analysis of the 10 selected key genes (ABCB1, AKR1C3, EIF2AK2, ELANE, FKBP5, GADD45A, IFIH1, LCN2, NPC1, SCO2) regarding their expression patterns and interactions in both the training and test sets. As shown in **Figure 5A**, AKR1C3, EIF2AK2, and NPC1 were downregulated in SLE, whereas ELANE, FKBP5, GADD45A, IFIH1, LCN2, and SCO2 were upregulated. Further validation in the test sets revealed that, except for ELANE, FKBP5, GADD45A, and LCN2, all other genes showed significant differences in both Test Set 1 and Test Set 2 (**Figures 5B,C**). Protein–protein interaction analysis indicated how these proteins interrelate (**Figure 5D**), and subsequent gene correlation analysis revealed significant associations among them, with IFIH1 and EIF2AK2 exhibiting the strongest correlation (r = 0.83) (**Figure 5E**). Additional ROC analysis assessed the predictive performance of these genes in the validation sets, yielding AUC values of 0.979 for SCO2, 0.960 for EIF2AK2, 0.939 for IFIH1, 0.886 for ABCB1, 0.863 for NPC1, 0.713 for AKR1C3, 0.677 for LCN2, 0.643 for GADD45A, 0.635 for FKBP5, and 0.540 for ELANE (**Figure 5F**).

### 3.5 Immune infiltration and the relationships between key genes and immune cells

**Figure 6:**
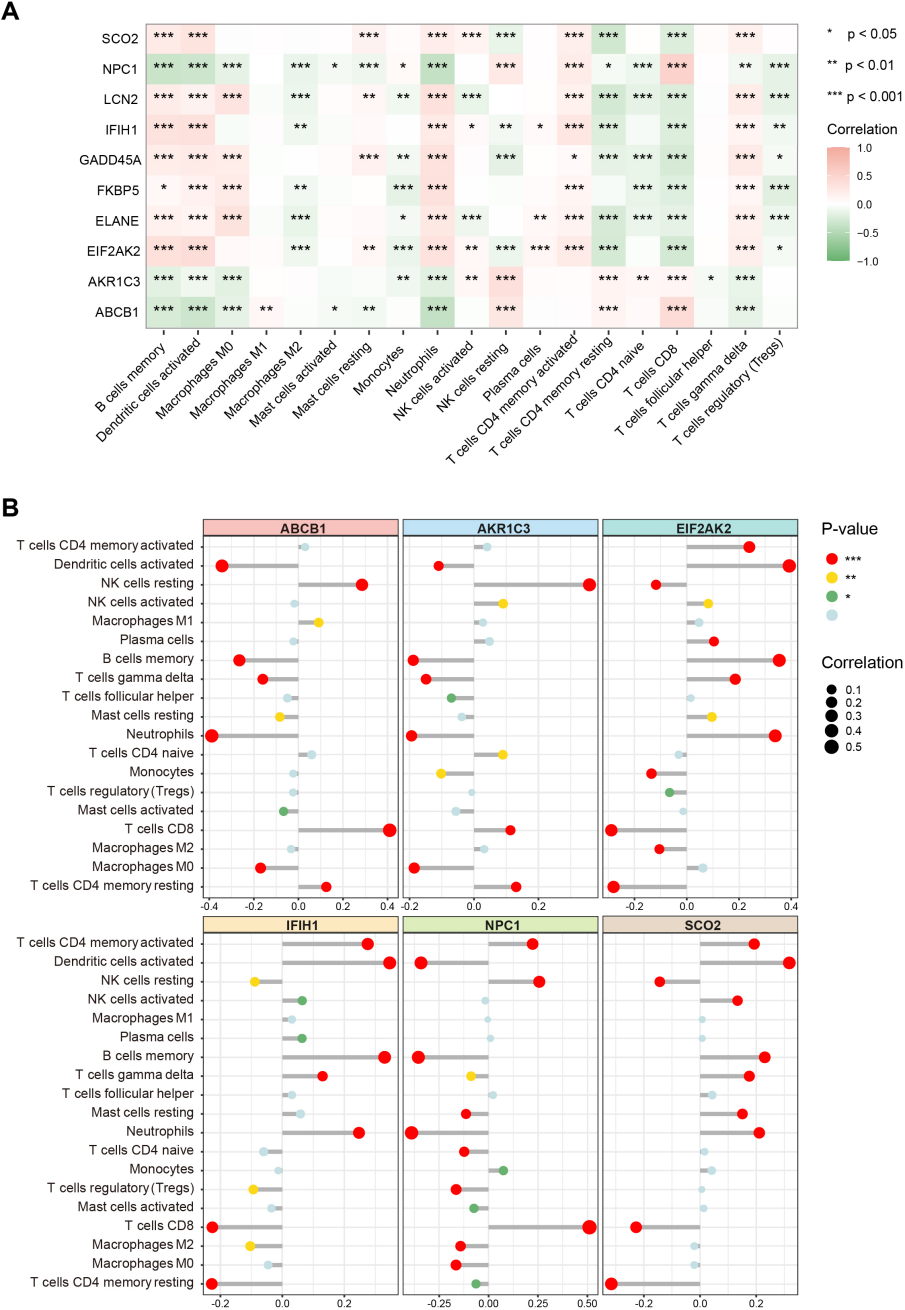
Associations of key genes with immune cell infiltration. **(A)** Heatmap showing correlations between the 10 key genes and various immune cell types. Color ranges from green to red, indicating positive to negative correlations, with color intensity reflecting the absolute value of the correlation coefficient. **(B)** Lollipop chart displaying the magnitude and significance of correlations between six major key genes and different immune cells. (*P < 0.05, **P < 0.01, ***P < 0.001).

To further elucidate the associations between these key genes and immune cells, we performed immune infiltration analysis on the target dataset. The results indicated extensive and significant correlations between these genes and various immune cell types (**Figure 6A**). Subsequently, six of the main key genes were selected for more detailed immune infiltration evaluation (**Figure 6B**). The results showed that each gene displayed distinct infiltration patterns and expression levels across different immune cell types, suggesting that these genes may play pivotal roles in SLE immunopathogenesis by regulating immune cell activation or differentiation.

### 3.6 Single-cell data expression

**Figure 7:**
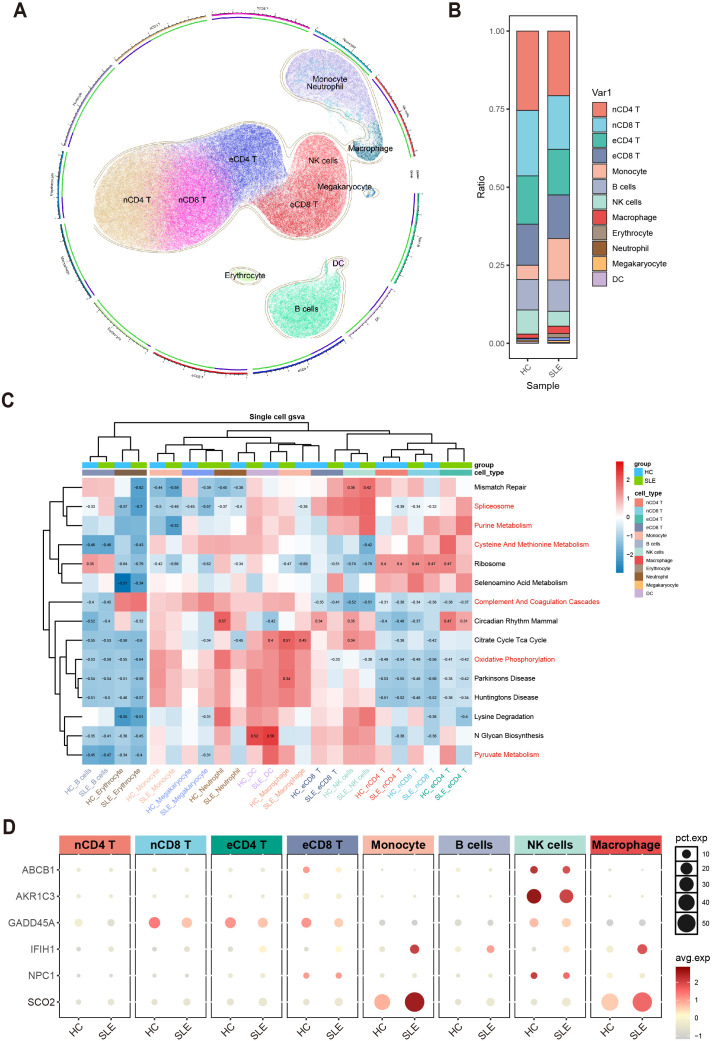
Single-cell–level immune cell distribution, key metabolic pathway enrichment, and differential expression of key genes in SLE versus healthy controls. **(A)** UMAP clustering of integrated single-cell transcriptomic data, where distinct colors and regions represent different cell types. **(B)** Stacked bar plot showing differences in the relative distribution of cell types between the SLE group and healthy control group. **(C)** Heatmap of GSVA analysis results at the single-cell level. **(D)** Bubble plot depicting the expression of six major key genes across different cell types and groups (HC, SLE). The size of each bubble indicates the percentage of cells expressing the gene, and the bubble color intensity reflects the average expression level.

To further clarify, at the single-cell level, the differences in immune cells and key gene expression between SLE patients and HCs, we performed UMAP clustering on the integrated PBMCs single-cell transcriptomic data. The analysis classified all cells into 11 clusters, which were annotated based on highly variable genes as monocytes, macrophages, naive CD4^+^ T cells (nCD4 T), naive CD8^+^ T cells (nCD8 T), effector memory CD4^+^ T cells (eCD4 T), effector memory CD8^+^ T cells (eCD8 T), B cells, NK cells, erythrocytes, neutrophils, megakaryocytes, and dendritic cells (DCs) (**Figure 7A**). A stacked bar chart (**Figure 7B**) showed notable differences in the distribution of these cell types between SLE patients and HCs, with naive CD4^+^ T cells, naive CD8^+^ T cells, effector memory CD4^+^ T cells, effector memory CD8^+^ T cells, monocytes, B cells, NK cells, and macrophages accounting for the majority of total cells. Of particular note, the proportions of naive CD4^+^ T cells and NK cells significantly decreased in the SLE group, whereas the proportions of monocytes and macrophages were markedly elevated.

Meanwhile, examining the activity of each immune cell type in key metabolic pathways (**Figure 7C**) revealed certain heterogeneity in their responses. Notably, changes in monocytes and macrophages within these critical pathways were highly consistent with the GSVA results from earlier transcriptomic analysis, further suggesting their potential significance in the metabolic dysregulation of SLE.

Additionally, assessing the expression profiles of the aforementioned key genes in various cell types revealed that ABCB1, AKR1C3, and NPC1 were primarily expressed in NK cells and downregulated in SLE; IFIH1 and SCO2 were mainly expressed in monocytes and macrophages and were upregulated in SLE; and GADD45A was mainly expressed in naive CD8^+^ T cells and effector memory CD8^+^ T cells, exhibiting downregulation in SLE (**Figure 7D**). These findings suggest a strong link between shifts in SLE-associated cellular subpopulations and the expression patterns of key genes.

### 3.7 RT-qPCR validation of major key genes

**Figure 8:**
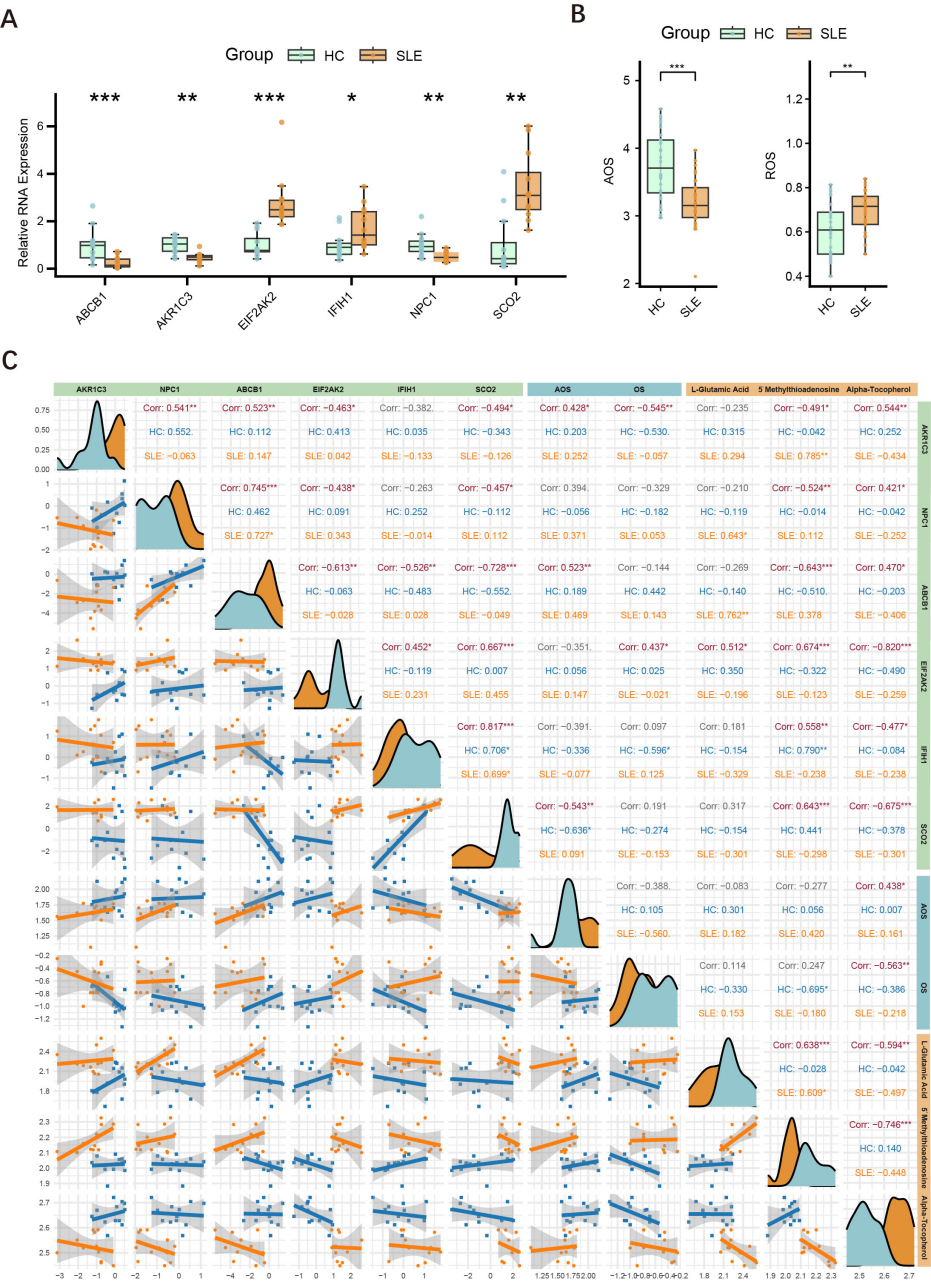
Expression of major key genes in SLE and HCs, and correlations with OS and metabolites. **(A)** Relative expression levels of six key genes (ABCB1, AKR1C3, EIF2AK2, IFIH1, NPC1, SCO2) in PBMCs measured by RT-qPCR. Asterisks indicate statistical significance. **(B)** Serum levels of AOS and ROS in the SLE and HC groups. **(C)** Correlation graph illustrating the relationships among the six key genes (AKR1C3, NPC1, ABCB1, EIF2AK2, IFIH1, SCO2), ASL, ROS, and three key metabolites (L-glutamate, 5′-methylthioadenosine, α-tocopherol) in the HC and SLE groups. Density curves appear along the diagonal; scatter plots with regression lines are shown below the diagonal; correlation coefficients (Corr) and significance levels are shown above. (*P < 0.05, **P < 0.01, ***P < 0.001).

To further validate the differential expression of major key genes in SLE patients and HCs, we performed RT-qPCR on PBMCs from 12 SLE patients and 12 HCs. Compared with the HC group, ABCB1, AKR1C3, and NPC1 were significantly downregulated in SLE patients, whereas EIF2AK2, IFIH1, and SCO2 were significantly upregulated (**Figure 8A**).

### 3.8 OS and AOS levels in SLE patients

Building upon the initial group of 12 SLE patients and 12 HCs, we further selected serum samples from an additional 30 SLE patients and 30 HCs to measure OS and AOS levels, with H_2_O_2_ serving as the primary indicator of OS. The results demonstrated that SLE patients had significantly higher OS levels and markedly lower AOS levels (**Figure 8B**), suggesting a redox imbalance in the pathogenesis of SLE.

### 3.9 Interconnections among key genes, OS, and related metabolites

Drawing on the findings in Sections 4.2 and 4.8, we conducted an integrated association analysis of key genes, OS, and related metabolites. As shown in **Figure 8C**, correlations among six key genes (AKR1C3, NPC1, ABCB1, EIF2AK2, IFIH1, SCO2), AOS levels, ROS levels, and three key metabolites (L-glutamate, 5′-methylthioadenosine, α-tocopherol) were evaluated in both HC and SLE groups. The six key genes were significantly correlated with one another, consistent with the findings in Section 4.4. Specifically, AKR1C3 and ABCB1 were positively correlated with AOS levels, whereas SCO2 was negatively correlated with AOS levels, and AKR1C3 was negatively correlated with OS levels, whereas EIF2AK2 was positively correlated with OS levels. Thus, AKR1C3, which associates with both OS and AOS, likely plays a pivotal role in the mechanisms of OS and AOS during SLE progression. Notably, the metabolite α-tocopherol not only correlated with other OS-related metabolites but also showed significant associations with these six genes and both OS and AOS levels, indicating that α-tocopherol may exert an important influence in the redox imbalance and key gene regulation of SLE.

## 4 Discussion

Metabolic patterns of immune cells under inflammatory conditions are closely related to OS levels. Upon T-cell activation, glycolysis is enhanced via the mTOR and HIF1α pathways, whereas B cells rely more on mitochondrial function and oxidative metabolism during plasma cell differentiation. Meanwhile, ROS can also modulate immune cell signaling pathways such as NF-κB and HIF-1α, thereby regulating both the intensity and duration of inflammatory responses (31). Notably, recent studies suggest that such redox-sensitive signaling processes may be spatially organized within lipid rafts—specialized membrane microdomains rich in cholesterol and sphingolipids (32). Lipid rafts facilitate the clustering of receptors and signaling molecules, including NF-κB and PI3K, and may serve as a structural platform linking OS with immune metabolic reprogramming (33). In recent years, interventions targeting OS and cellular metabolism have emerged as promising therapeutic approaches for SLE. Various antioxidant agents—including luteolin, itaconic acid, and ethyl pyruvate—have been reported to ameliorate clinical symptoms in SLE (34–36). Nevertheless, the mechanisms of interaction between OS and metabolic processes among different immune cells remain insufficiently elucidated, necessitating more systematic studies to clarify their role in disease pathogenesis and progression.

In this study, EIF2AK2 and IFIH1, two OS-related key genes highly associated with SLE, displayed a significant positive correlation. This correlation may exert a critical influence on SLE pathogenesis through the JAK-STAT pathway. EIF2AK2 encodes the protein kinase R (PKR), a core regulator of the stress response that is highly sensitive to OS signals and regulated by STAT3. PKR interacts with multiple inflammasomes (e.g., NLRP3, NLRP1, NLRC4, AIM2) to govern cytokine release (37–39). Previous work has shown that circRNA levels in PBMCs from SLE patients are markedly reduced, potentially causing excessive PKR activation, which exacerbates OS and the inflammatory response (40). Meanwhile, IFIH1 is an intracellular receptor that recognizes viral RNA and induces type I interferons and other inflammatory factors. Upon activation during infection, IFIH1 can also promote STAT1 transcription, facilitating M1 polarization in macrophages and intensifying inflammation (41–43). Hence, EIF2AK2 and IFIH1 may functionally contribute to the pathogenesis and persistence of SLE by jointly driving oxidative stress imbalance and sustained chronic inflammation through the regulation of the JAK-STAT pathway and immune-inflammatory networks.

ABCB1 is a member of the ATP-binding cassette transporter family. It encodes P-glycoprotein, whose primary function is to expel intracellular toxins via ATP-driven transport (44). In SLE patients, ABCB1 expression is decreased in both memory and activated B cells, possibly contributing to B-cell dysfunction and disease activity (45, 46). Notably, ABCB1 plays a crucial role in tumor drug resistance, where ROS can suppress P-glycoprotein function or alter its expression, indirectly affecting the cytotoxicity of chemotherapeutics (44, 47). Moreover, under hypoxic conditions, increased oxidative phosphorylation can further activate the Hif1α-ABCB1 axis, thereby enhancing P-glycoprotein function (48). Importantly, P-glycoprotein can also be transcriptionally upregulated in response to oxidative stress via stress-responsive factors such as Nrf2 and NF-κB, and contributes to maintaining redox homeostasis by actively exporting lipid peroxidation products and other ROS-generating compounds (49). Therefore, reduced ABCB1 expression may not only be a downstream consequence of oxidative stress, but also exacerbate redox imbalance by impairing ROS clearance. This evidence supports a potential causal role of ABCB1 dysregulation in sustaining oxidative stress in SLE. In the present study, ABCB1 expression was similarly downregulated in SLE patients’ B cells, though this decrease was more pronounced in NK cells; concurrently, oxidative phosphorylation in NK cells was also markedly reduced. Since oxidative phosphorylation is the principal source of ATP, compromised NK-cell function in SLE may stem from insufficient energy production, which in turn could lead to reduced ABCB1 expression. These observations underscore the need for further investigation into the precise role of ABCB1 in SLE pathogenesis, particularly regarding its functional and signaling interactions in B cells and NK cells.

This study is the first to report a strong association between AKR1C3 and the OS/AOS balance in SLE patients, indicating that AKR1C3 is closely linked to OS and AOS levels in SLE. AKR1C3 can catalyze the production of testosterone and dihydrotestosterone, thereby activating androgen receptor signaling (50). It is also an integral component of the cellular defense system against OS, functioning under high-OS conditions as an adaptive mechanism to mitigate ROS-induced cellular damage and help maintain proliferative capacity and metabolic function (51). Notably, studies have shown that AKR1C3 overexpression can activate the Keap1–Nrf2–ARE pathway, thereby enhancing the expression of antioxidant enzymes such as glutathione peroxidases (GPXs) and glutathione synthesis enzymes, ultimately reducing intracellular ROS levels. This positive feedback mechanism allows AKR1C3 to act not only as a downstream target of Nrf2 but also as an upstream enhancer of the Nrf2-mediated antioxidant response. In the context of SLE, which is characterized by chronic oxidative stress, the upregulation of AKR1C3 may serve as an adaptive mechanism to enhance antioxidant defenses and limit inflammation-induced oxidative injury (52).These findings provide a starting point for subsequent studies on the regulatory pathways and potential therapeutic value of AKR1C3 in SLE pathogenesis.

We further discovered that the key metabolite α-tocopherol is closely connected to the redox state in SLE and to the levels of key genes. α-Tocopherol, a fat-soluble vitamin with potent antioxidant properties, curtails lipid peroxidation by scavenging ROS, thereby mitigating cell damage induced by free radicals (28). In the present study, it also exhibited strong correlations with critical genes and metabolites. One investigation demonstrated that the α-tocopherol derivative α-T-13′-COOH exerts regulatory effects on multiple antioxidant pathways, particularly influencing the expression and activity of enzymes such as superoxide dismutase (SOD), glutathione S-transferase (GST), and catalase (CAT), thereby bolstering a cell’s ability to clear ROS. Additionally, α-tocopherol can modulate redox-sensitive genes (e.g., Nrf2, Hmox1), prompting cells to adapt to OS and mitigate oxidative damage. In RAW264.7 mouse macrophages, α-T-13′-COOH quickly induces ROS generation, but the cells subsequently adapt through enhanced antioxidant defenses (53). This evidence further underscores the critical role of α-tocopherol in redox homeostasis and immune regulation in SLE.

In SLE, anti-RLIP76 C-terminal antibodies are significantly elevated and can induce endothelial cell apoptosis via OS, leading to vascular dysfunction; however, antioxidant treatments such as α-tocopherol can effectively diminish OS and cell apoptosis driven by these antibodies (54). Furthermore, α-tocopherol indirectly regulates ABCB1 expression and function by reducing ROS levels, thereby preserving the integrity of the blood–brain barrier and preventing the buildup of toxic substances (55). These antioxidant effects may have potential utility in safeguarding the blood–brain barrier and slowing disease progression. Hence, α-tocopherol plays a crucial role in modulating OS, maintaining cellular homeostasis, and ameliorating immune dysfunction.

Notably, PCA based on LC-MS metabolomic data revealed substantial inter-individual heterogeneity among SLE samples, consistent with previous findings (56). Further inspection showed that the SLE samples formed two relatively distinct subgroups in principal component space, potentially driven by differences in disease activity levels (57). Given that OS status is strongly influenced by inflammatory burden and immune activation, metabolic profiles may vary significantly across different activity states. This clinical heterogeneity could critically impact the interpretation of OS-related features. Although the present study primarily focused on population-level analyses, the observed subgroup differentiation highlights the need for future stratification studies that integrate comprehensive clinical data. As larger cohorts and more detailed clinical information become available, it may be possible to further elucidate OS characteristics across SLE subtypes, thereby providing a theoretical basis for personalized therapeutic strategies.

Collectively, this study systematically characterized OS–related features in SLE, identifying several key genes and metabolites with diagnostic and therapeutic potential. These findings were validated across multiple omics layers and datasets, and linked to distinct immune cell subsets. Compared with previous studies that focused solely on transcriptomic data and machine-learning–based biomarker screening (58–60), our study presents three notable innovations. First, we employed a multi-omics framework that integrates bulk Transcriptomic, single-cell transcriptomics, and LC–MS–based metabolomics to achieve cross-validation. Second, we focused specifically on OS and AOS balance, a disease-relevant mechanism not emphasized in prior work. Third, we validated key genes through both PBMC-based qPCR and metabolomic profiling, constructing a gene–metabolite–immune network with translational implications. These features distinguish our study from prior literature and support its broader relevance to SLE pathogenesis.

Nonetheless, several limitations should be acknowledged. Further mechanistic studies are needed to elucidate the precise roles of key genes and the effects of targeting these genes or metabolites on disease pathology. Although the sample size for LC-MS analysis was moderate, the significant metabolic alterations and clear separation between groups in multivariate analyses suggest that the data were sufficient to capture key metabolic signatures in SLE. Future studies with larger cohorts will help validate these findings. In addition, some heterogeneity in the public datasets remains a concern—for example, all patients in GSE61635 were anti-RNP positive, and both GSE65391 and GSE135779 included pediatric samples, which may influence generalizability. We addressed this issue by combining multiple datasets and validating findings across data types to ensure robustness. Notably, GSE65391 contains many samples from adolescents approaching adulthood and has overall high data quality, making it appropriate for model construction (61). Even so, we acknowledge that such heterogeneity may influence generalizability. Our findings should thus be interpreted as identifying shared OS-related signals in SLE, rather than mechanisms specific to age group or antibody subtype.

## 5 Conclusions

By integrating multi-omics data analyses, this study systematically investigated key metabolic pathways and differential metabolites in SLE patients, demonstrating a close association between SLE and OS. Through a comprehensive screening and rigorous validation using five machine learning algorithms, six OS-related key genes highly correlated with SLE (EIF2AK2, AKR1C3, ABCB1, NPC1, IFIH1, SCO2) were ultimately identified and further confirmed in additional datasets for their predictive performance and associations with immune cell subsets. Subsequently, RT-qPCR experiments and measurements of OS and AOS levels revealed abnormal expression of these genes in SLE patient PBMCs and highlighted a dysregulated redox balance in SLE patients. The tight interconnections among key genes, redox status, and major metabolites, as uncovered in this study, provide essential theoretical support for characterizing the OS features of SLE.

## Data Availability

The datasets presented in this study can be found in online repositories. The names of the repository/repositories and accession number(s) can be found in the article/**Supplementary Material**.

## References

[1] Kiriakidou M Ching CL. Systemic lupus erythematosus. Ann Internal Med. (2020) 172:ITC81–96. doi: 10.7326/AITC202006020 32479157

[2] Luan H Gu W Li H Wang Z Lu L Ke M. Serum metabolomic and lipidomic profiling identifies diagnostic biomarkers for seropositive and seronegative rheumatoid arthritis patients. J Transl Med. (2021) 19:500. doi: 10.1186/s12967-021-03169-7 34876179 PMC8650414

[3] Li S Ding H Qi Z Yang J Huang J Huang L. Serum metabolic fingerprints characterize systemic lupus erythematosus. Advanced Sci. (2024) 11:2304610. doi: 10.1002/advs.202304610 PMC10787061 37953381

[4] Gautam P Kaur G Tandon A Sharma A Bhatnagar A. Altered redox regulation by Nrf2-Keap1 system in dendritic cells of systemic lupus erythematosus patients. Lupus. (2020) 29:1544–55. doi: 10.1177/0961203320950022 32811277

[5] Liu L De Leeuw K Arends S Doornbos-van Der Meer B Bulthuis MLC Van Goor H. Biomarkers of oxidative stress in systemic lupus erythematosus patients with active nephritis. Antioxidants. (2023) 12:1627. doi: 10.3390/antiox12081627 37627622 PMC10451241

[6] Cimmino TP Ammendola R Cattaneo F Esposito G. NOX dependent ROS generation and cell metabolism. Int J Mol Sci. (2023) 24:2086. doi: 10.3390/ijms24032086 36768405 PMC9916913

[7] Corkey BE Deeney JT. The redox communication network as a regulator of metabolism. Front Physiol. (2020) 11:567796. doi: 10.3389/fphys.2020.567796 33178037 PMC7593883

[8] Boese AC Kang S. Mitochondrial metabolism-mediated redox regulation in cancer progression. Redox Biol. (2021) 42:101870. doi: 10.1016/j.redox.2021.101870 33509708 PMC8113029

[9] Kwon DH Cha H-J Lee H Hong S-H Park C Park S-H. Protective Effect of Glutathione against Oxidative Stress-induced Cytotoxicity in RAW 264.7 Macrophages through Activating the Nuclear Factor Erythroid 2-Related Factor-2/Heme Oxygenase-1 Pathway. Antioxidants. (2019) 8:82. doi: 10.3390/antiox8040082 30939721 PMC6523540

[10] Yin Y Choi S-C Xu Z Perry DJ Seay H Croker BP. Normalization of CD4^+^ T cell metabolism reverses lupus. Sci Transl Med. (2015) 7:27418–8. doi: 10.1126/scitranslmed.aaa0835 PMC5292723 25673763

[11] Shan J Jin H Xu Y. T cell metabolism: A new perspective on th17/treg cell imbalance in systemic lupus erythematosus. Front Immunol. (2020) 11:1027. doi: 10.3389/fimmu.2020.01027 32528480 PMC7257669

[12] Sharabi A Tsokos GC. T cell metabolism: new insights in systemic lupus erythematosus pathogenesis and therapy. Nat Rev Rheumatol. (2020) 16:100–12. doi: 10.1038/s41584-019-0356-x 31949287

[13] Scherlinger M Pan W Hisada R Boulougoura A Yoshida N Vukelic M. Phosphofructokinase P fine-tunes T regulatory cell metabolism, function, and stability in systemic autoimmunity. Sci Adv. (2022) 8:eadc9657. doi: 10.1126/sciadv.adc9657 36449620 PMC9710877

[14] Montano EN Bose M Huo L Tumurkhuu G De Los Santos G Simental B. α-Ketoglutarate-Dependent KDM6 Histone Demethylases and Interferon-Stimulated Gene Expression in Lupus. Arthritis Rheumatol. (2024) 76:396–410. doi: 10.1002/art.42724 37800478 PMC10922114

[15] Xu S Li X Zhang S Qi C Zhang Z Ma R. Oxidative stress gene expression, DNA methylation, and gut microbiota interaction trigger crohn’s disease: a multi-omics mendelian randomization study. BMC Med. (2023) 21:179. doi: 10.1186/s12916-023-02878-8 37170220 PMC10173549

[16] Fan J Cao S Chen M Yao Q Zhang X Du S. Investigating the AC079305/DUSP1 axis as oxidative stress-related signatures and immune infiltration characteristics in ischemic stroke. Oxid Med Cell Longevity. (2022) 2022:8432352. doi: 10.1155/2022/8432352 PMC9213160 35746962

[17] Wu X Wei D Zhou Y Cao Q Han G Han E. Pesticide exposures and 10-year atherosclerotic cardiovascular disease risk: Integrated epidemiological and bioinformatics analysis. J Hazard Mater. (2025) 485:136835. doi: 10.1016/j.jhazmat.2024.136835 39673955

[18] Kanehisa M Goto S Sato Y Furumichi M Tanabe M. KEGG for integration and interpretation of large-scale molecular data sets. Nucleic Acids Res. (2012) 40:D109–114. doi: 10.1093/nar/gkr988 PMC3245020 22080510

[19] Newman AM Liu CL Green MR Gentles AJ Feng W Xu Y. Robust enumeration of cell subsets from tissue expression profiles. Nat Methods. (2015) 12:453–7. doi: 10.1038/nmeth.3337 PMC4739640 25822800

[20] Hochberg MC. Updating the american college of rheumatology revised criteria for the classification of systemic lupus erythematosus. Arthritis Rheum. (1997) 40:1725. doi: 10.1002/art.1780400928 9324032

[21] Wu H Gonzalez Villalobos R Yao X Reilly D Chen T Rankin M. Mapping the single-cell transcriptomic response of murine diabetic kidney disease to therapies. Cell Metab. (2022) 34:1064–1078.e6. doi: 10.1016/j.cmet.2022.05.010 35709763 PMC9262852

[22] Roy JG McElhaney JE Verschoor CP. Reliable reference genes for the quantification of mRNA in human T-cells and PBMCs stimulated with live influenza virus. BMC Immunol. (2020) 21:4. doi: 10.1186/s12865-020-0334-8 32005148 PMC6995044

[23] Sies H. Oxidative stress: a concept in redox biology and medicine. Redox Biol. (2015) 4:180–3. doi: 10.1016/j.redox.2015.01.002 PMC4309861 25588755

[24] Martínez Y Li X Liu G Bin P Yan W Más D. The role of methionine on metabolism, oxidative stress, and diseases. Amino Acids. (2017) 49:2091–8. doi: 10.1007/s00726-017-2494-2 28929442

[25] Hayes JD Dinkova-Kostova AT. The Nrf2 regulatory network provides an interface between redox and intermediary metabolism. Trends Biochem Sci. (2014) 39:199–218. doi: 10.1016/j.tibs.2014.02.002 24647116

[26] Torres-Odio S Key J Hoepken H-H Canet-Pons J Valek L Roller B. Progression of pathology in PINK1-deficient mouse brain from splicing via ubiquitination, ER stress, and mitophagy changes to neuroinflammation. J Neuroinflamm. (2017) 14:154. doi: 10.1186/s12974-017-0928-0 PMC5541666 28768533

[27] Loukovaara S Nurkkala H Tamene F Gucciardo E Liu X Repo P. Quantitative proteomics analysis of vitreous humor from diabetic retinopathy patients. J Proteome Res. (2015) 14:5131–43. doi: 10.1021/acs.jproteome.5b00900 26490944

[28] Pisoschi AM Pop A. The role of antioxidants in the chemistry of oxidative stress: A review. Eur J Medicinal Chem. (2015) 97:55–74. doi: 10.1016/j.ejmech.2015.04.040 25942353

[29] Ma S Song W Xu Y Si X Lv S Zhang Y. Rationally designed polymer conjugate for tumor-specific amplification of oxidative stress and boosting antitumor immunity. Nano Lett. (2020) 20:2514–21. doi: 10.1021/acs.nanolett.9b05265 32109068

[30] Yuan L Liu H Liu X Zhang X Wu J Wang Y. Epigenetic modification of H3K4 and oxidative stress are involved in MC-LR-induced apoptosis in testicular cells of SD rats. Environ Toxicol. (2020) 35:277–91. doi: 10.1002/tox.22865 31691492

[31] Morris G Gevezova M Sarafian V Maes M. Redox regulation of the immune response. Cell Mol Immunol. (2022) 19:1079–101. doi: 10.1038/s41423-022-00902-0 PMC9508259 36056148

[32] Isik OA Cizmecioglu O. Rafting on the plasma membrane: lipid rafts in signaling and disease. Adv Exp Med Biol. (2023) 1436:87–108. doi: 10.1007/5584_2022_759 36648750

[33] Muzammil K Sabah Ghnim Z Saeed Gataa I Fawzi Al-Hussainy A Ali Soud N Adil M. NRF2-mediated regulation of lipid pathways in viral infection. Mol Aspects Med. (2024) 97:101279. doi: 10.1016/j.mam.2024.101279 38772081

[34] Ding T Yi T Li Y Zhang W Wang X Liu J. Luteolin attenuates lupus nephritis by regulating macrophage oxidative stress via HIF-1α pathway. Eur J Pharmacol. (2023) 953:175823. doi: 10.1016/j.ejphar.2023.175823 37263402

[35] Blanco LP Patino-Martinez E Nakabo S Zhang M Pedersen HL Wang X. Modulation of the itaconate pathway attenuates murine lupus. Arthritis Rheumatol. (2022) 74:1971–83. doi: 10.1002/art.42284 PMC11337117 35791960

[36] Koprivica I Djedovic N Stojanović I Miljković Đ. Ethyl pyruvate, a versatile protector in inflammation and autoimmunity. Inflammation Res. (2022) 71:169–82. doi: 10.1007/s00011-021-01529-z PMC8742706 34999919

[37] Lu B Nakamura T Inouye K Li J Tang Y Lundbäck P. Novel role of PKR in inflammasome activation and HMGB1 release. Nature. (2012) 488:670–4. doi: 10.1038/nature11290 PMC4163918 22801494

[38] Meyer C Garzia A Mazzola M Gerstberger S Molina H Tuschl T. The TIA1 RNA-binding protein family regulates EIF2AK2-mediated stress response and cell cycle progression. Mol Cell. (2018) 69:622–635.e6. doi: 10.1016/j.molcel.2018.01.011 29429924 PMC5816707

[39] You L Wang Z Li H Shou J Jing Z Xie J. The role of STAT3 in autophagy. Autophagy. (2015) 11:729–39. doi: 10.1080/15548627.2015.1017192 PMC4509450 25951043

[40] Liu C-X Li X Nan F Jiang S Gao X Guo S-K. Structure and degradation of circular RNAs regulate PKR activation in innate immunity. Cell. (2019) 177:865–880.e21. doi: 10.1016/j.cell.2019.03.046 31031002

[41] Molineros JE Maiti AK Sun C Looger LL Han S Kim-Howard X. Admixture mapping in lupus identifies multiple functional variants within IFIH1 associated with apoptosis, inflammation, and autoantibody production. PloS Genet. (2013) 9:e1003222. doi: 10.1371/journal.pgen.1003222 23441136 PMC3575474

[42] Wang A Kang X Wang J Zhang S. IFIH1/IRF1/STAT1 promotes sepsis associated inflammatory lung injury via activating macrophage M1 polarization. Int Immunopharmacol. (2023) 114:109478. doi: 10.1016/j.intimp.2022.109478 36462334 PMC9709523

[43] Zhang J Liu X Meng Y Wu H Wu Y Yang B. Autoimmune disease associated IFIH1 single nucleotide polymorphism related with IL-18 serum levels in Chinese systemic lupus erythematosus patients. Sci Rep. (2018) 8:9442. doi: 10.1038/s41598-018-27782-7 29930297 PMC6013496

[44] Robey RW Pluchino KM Hall MD Fojo AT Bates SE Gottesman MM. Revisiting the role of ABC transporters in multidrug-resistant cancer. Nat Rev Cancer. (2018) 18:452–64. doi: 10.1038/s41568-018-0005-8 PMC6622180 29643473

[45] Fleischer SJ Giesecke C Mei HE Lipsky PE Daridon C Dörner T. Increased frequency of a unique spleen tyrosine kinase bright memory B cell population in systemic lupus erythematosus. Arthritis Rheumatol. (2014) 66:3424–35. doi: 10.1002/art.38854 25156507

[46] Engle K Kumar G. Cancer multidrug-resistance reversal by ABCB1 inhibition: A recent update. Eur J Medicinal Chem. (2022) 239:114542. doi: 10.1016/j.ejmech.2022.114542 35751979

[47] Gajdács M Spengler G Sanmartín C Marć MA Handzlik J Domínguez-Álvarez E. Selenoesters and selenoanhydrides as novel multidrug resistance reversing agents: A confirmation study in a colon cancer MDR cell line. Bioorganic Medicinal Chem Lett. (2017) 27:797–802. doi: 10.1016/j.bmcl.2017.01.033 28126516

[48] Guan Q Wang X Cao D Li M Luo Z Mao X. Calcium phosphate-based nanoformulation selectively abolishes phenytoin resistance in epileptic neurons for ceasing seizures. Small. (2023) 19:2300395. doi: 10.1002/smll.202300395 37029709

[49] Shchulkin AV Abalenikhina YV Kosmachevskaya OV Topunov AF Yakusheva EN. Regulation of P-glycoprotein during oxidative stress. Antioxidants. (2024) 13:215. doi: 10.3390/antiox13020215 38397813 PMC10885963

[50] Paulukinas RD Mesaros CA Penning TM. Conversion of classical and 11-oxygenated androgens by insulin-induced AKR1C3 in a model of human PCOS adipocytes. Endocrinology. (2022) 163:bqac068. doi: 10.1210/endocr/bqac068 35560164 PMC9162389

[51] Zhang H Qian Y Zhang Y Zhou X Shen S Li J. Multi-omics analysis deciphers intercellular communication regulating oxidative stress to promote oral squamous cell carcinoma progression. NPJ Precis Onc. (2024) 8:272. doi: 10.1038/s41698-024-00764-x PMC11582705 39572698

[52] MacLeod AK Acosta-Jimenez L Coates PJ McMahon M Carey FA Honda T. Aldo-keto reductases are biomarkers of NRF2 activity and are co-ordinately overexpressed in non-small cell lung cancer. Br J Cancer. (2016) 115:1530–9. doi: 10.1038/bjc.2016.363 PMC5155360 27824809

[53] Liao S Börmel L Müller AK Gottschalk L Pritsch N Preisner LZ. α-tocopherol long-chain metabolite α-T-13′-COOH exhibits biphasic effects on cell viability, induces ROS-dependent DNA damage, and modulates redox status in murine RAW264.7 macrophages. Mol Nutr Food Res. (2024) 68:2400455. doi: 10.1002/mnfr.202400455 39548913 PMC11653165

[54] Margutti P Matarrese P Conti F Colasanti T Delunardo F Capozzi A. Autoantibodies to the C-terminal subunit of RLIP76 induce oxidative stress and endothelial cell apoptosis in immune-mediated vascular diseases and atherosclerosis. Blood. (2008) 111:4559–70. doi: 10.1182/blood-2007-05-092825 PMC2343593 17993611

[55] Sita G Hrelia P Tarozzi A Morroni F. P-glycoprotein (ABCB1) and oxidative stress: focus on alzheimer’s disease. Oxid Med Cell Longevity. (2017) 2017:7905486. doi: 10.1155/2017/7905486 PMC5727796 29317984

[56] He J Tang D Liu D Hong X Ma C Zheng F. Serum proteome and metabolome uncover novel biomarkers for the assessment of disease activity and diagnosing of systemic lupus erythematosus. Clin Immunol. (2023) 251:109330. doi: 10.1016/j.clim.2023.109330 37075949

[57] Liang J Han Z Feng J Xie F Luo W Chen H. Targeted metabolomics combined with machine learning to identify and validate new biomarkers for early SLE diagnosis and disease activity. Clin Immunol (orlando Fla). (2024) 264:110235. doi: 10.1016/j.clim.2024.110235 38710348

[58] Zhao X Duan L Cui D Xie J. Exploration of biomarkers for systemic lupus erythematosus by machine-learning analysis. BMC Immunol. (2023) 24:44. doi: 10.1186/s12865-023-00581-0 37950194 PMC10638835

[59] Lv J Chen L Wang X Gao Q Zhao L. Immune-relevant genes of systemic lupus erythematosus by transcriptome profiling analysis. Cytokine. (2022) 158:155975. doi: 10.1016/j.cyto.2022.155975 35964416

[60] Kong J Li L Zhimin L Yan J Ji D Chen Y. Potential protein biomarkers for systemic lupus erythematosus determined by bioinformatics analysis. Comput Biol Chem. (2019) 83:107135. doi: 10.1016/j.compbiolchem.2019.107135 31751880

[61] Banchereau R Hong S Cantarel B Baldwin N Baisch J Edens M. Personalized immunomonitoring uncovers molecular networks that stratify lupus patients. Cell. (2016) 165:551–65. doi: 10.1016/j.cell.2016.03.008 PMC5426482 27040498

