# Supplementary material for: Machine learning combined multi-omics analysis to explore key oxidative stress features in systemic lupus erythematosus
Source: Front Immunol. 2025 Jun 20;16:1567466. doi: 10.3389/fimmu.2025.1567466 (PMC12231464; doi:10.3389/fimmu.2025.1567466)
Supplement: Supplementary file 1 [file Table1.docx]

| **Supplementary table 1：Primer sequence** | |
| --- | --- |
| EIF2AK2 | F：AGAAGGCGGAGCGTGAAGTAAAAG |
|  | R：ATCCATCCCAACAGCCATTGTAGTG |
| AKR1C3 | F：GAGAAGTAAAGCTTTGGAGGTCACA |
|  | R：CAACCTGCTCCTCATTATTGTATAAATGA |
| ABCB1 | F：FGTCTGGACAAGCACTGAAAGATAAGA |
|  | R：CAACGGTTCGGAAGTTTTCTATTGC |
| NPC1 | F：GCACCTTTTACCATCACTCCTG |
|  | R：GGCCACAGACAATAGAGCAGT |
| IFIH1 | F：TCGAATGGGTATTCCACAGACG |
|  | R：GTGGCGACTGTCCTCTGAA |
| SCO2 | F：AGCAGCAAAAGCGAACAGAAG |
| GAPDH | R：GGCAGTGAGTGAAGCCAAAG  F：GGAGCGAGATCCCTCCAAAAT  R：GGCTGTTGTCATACTTCTCATGG |
